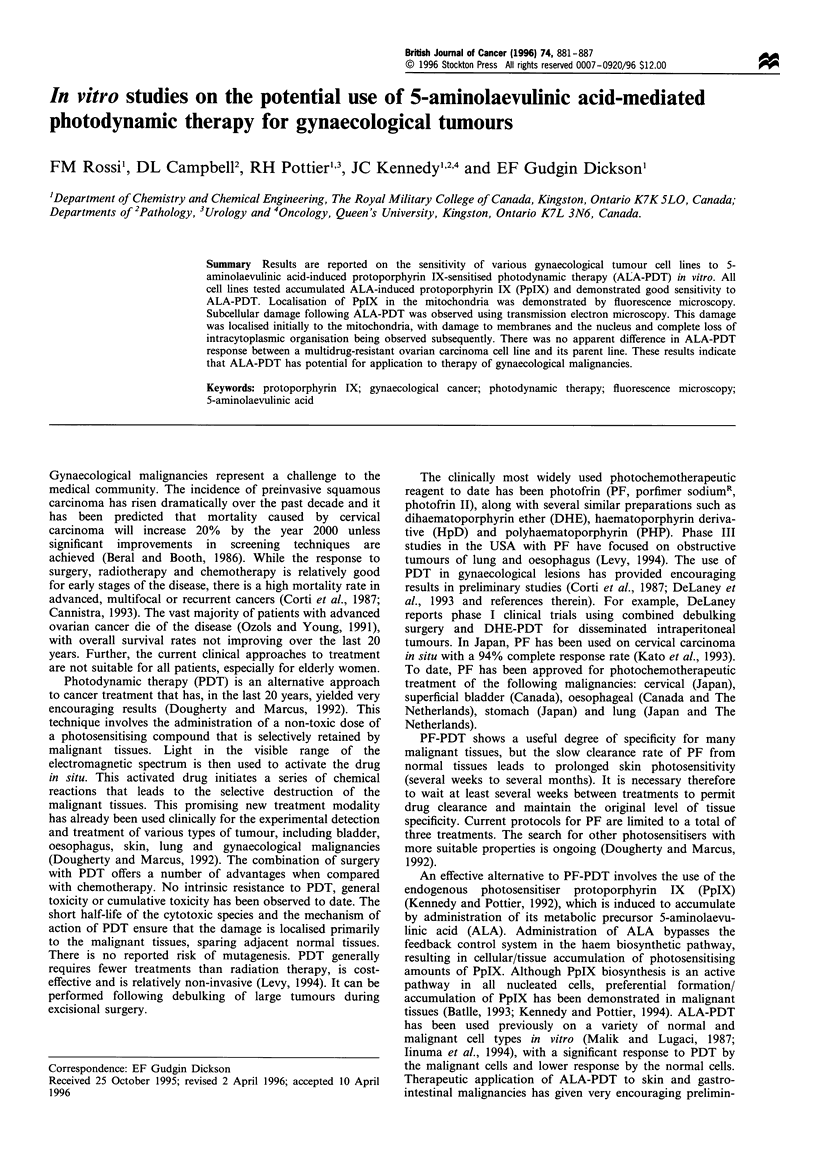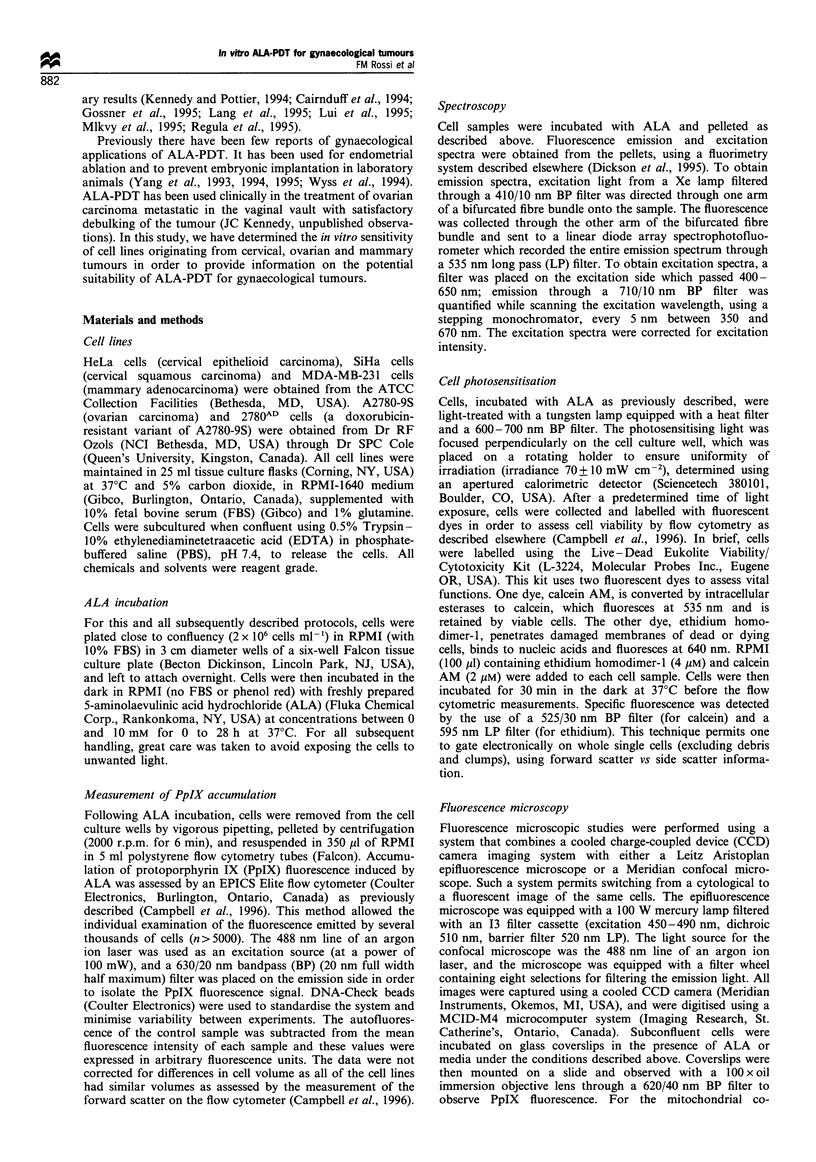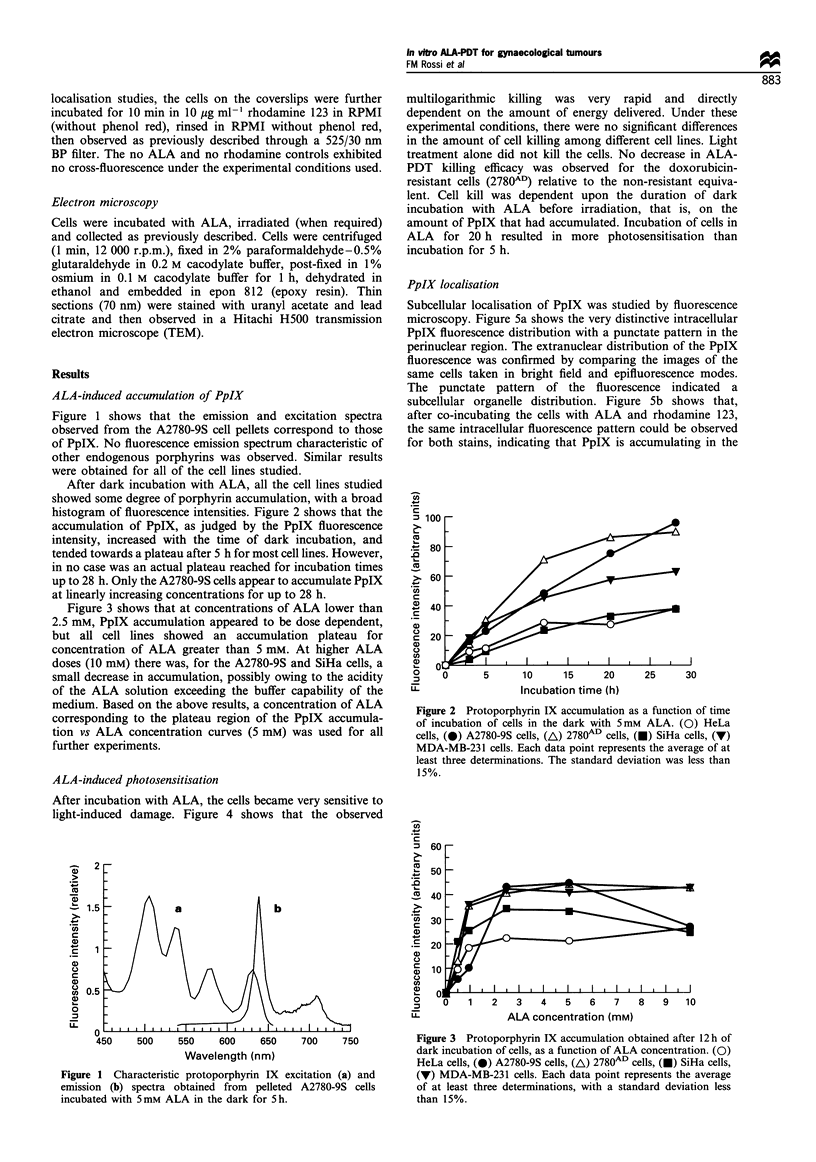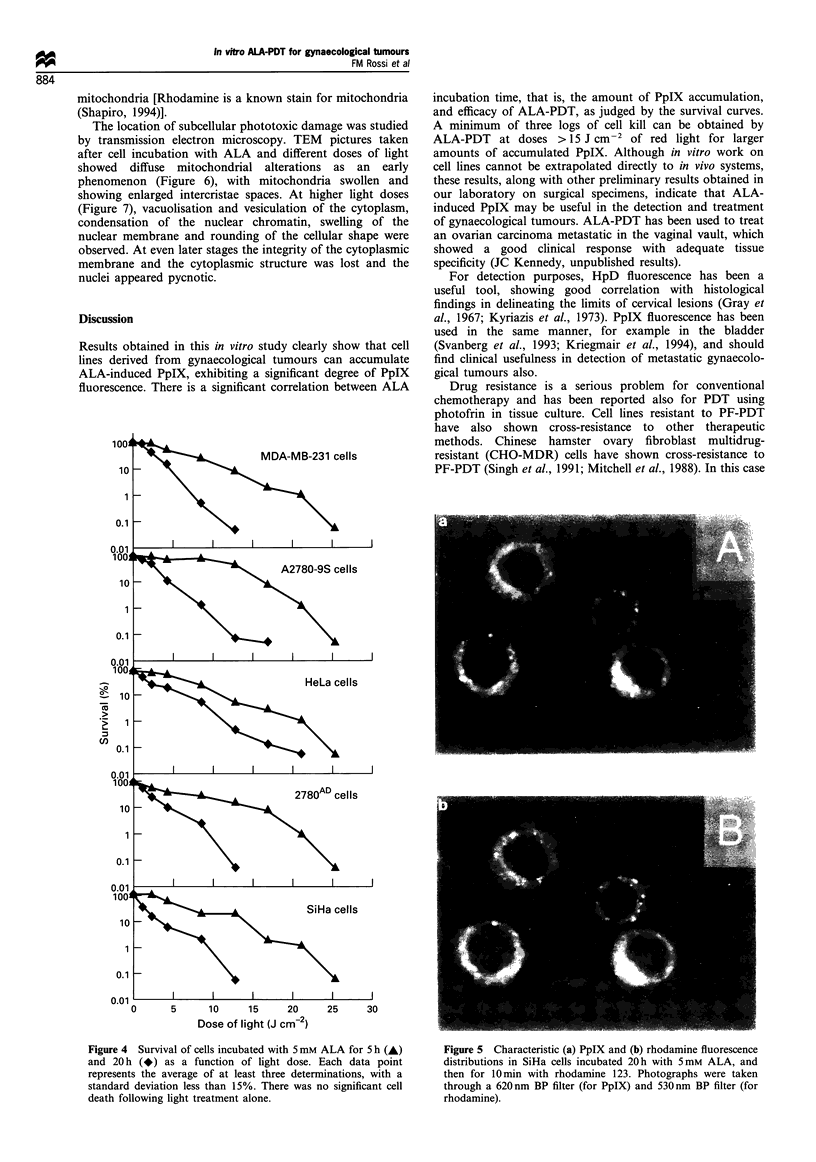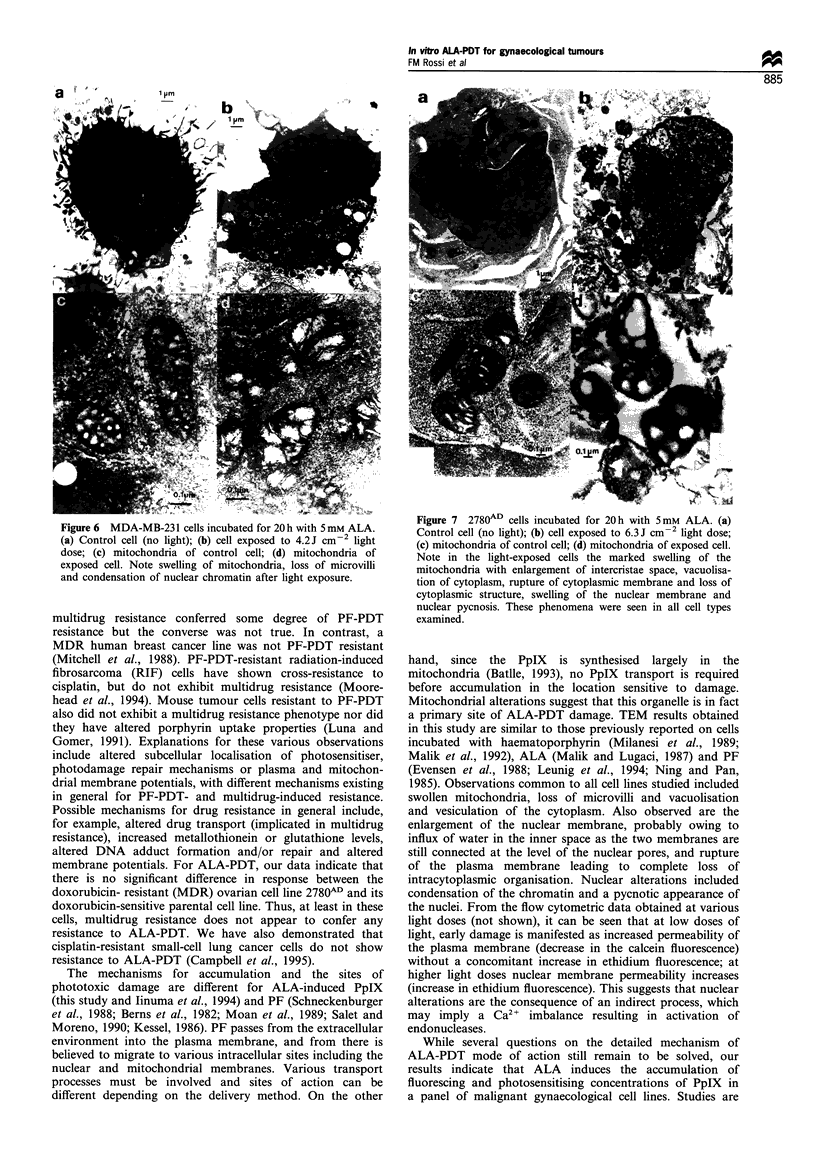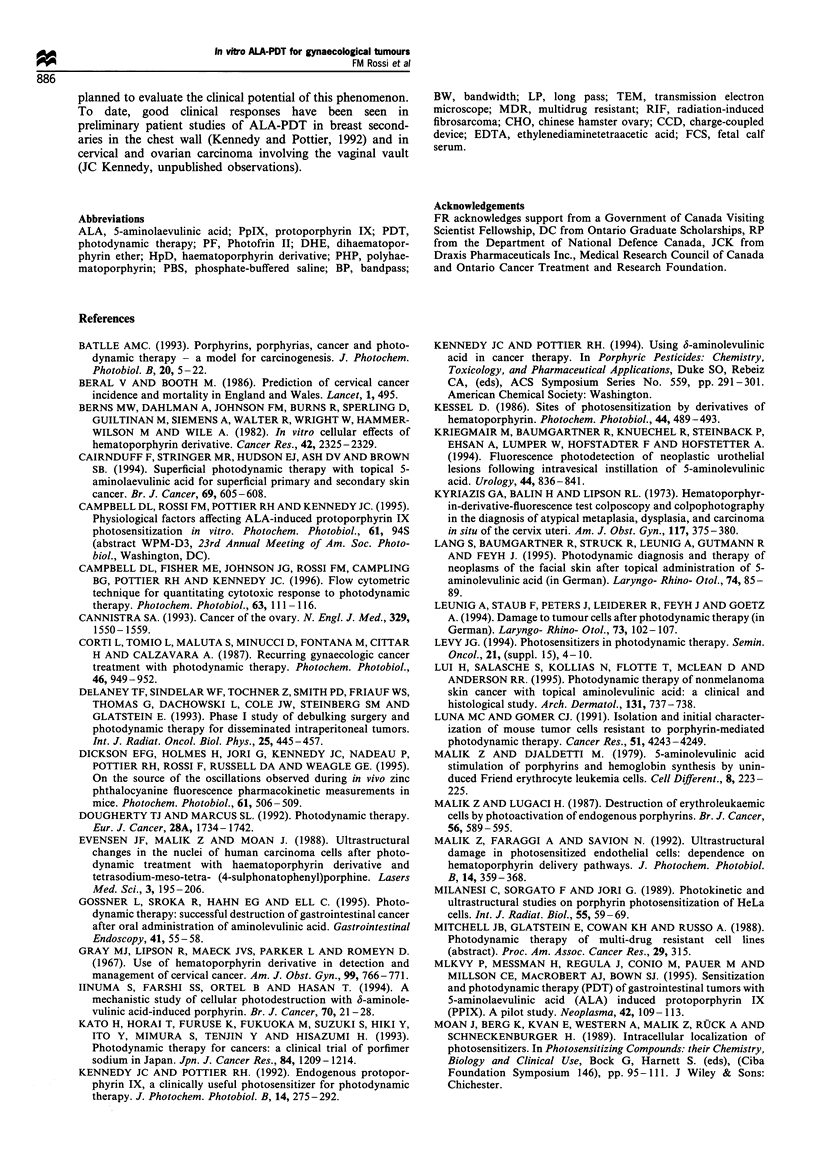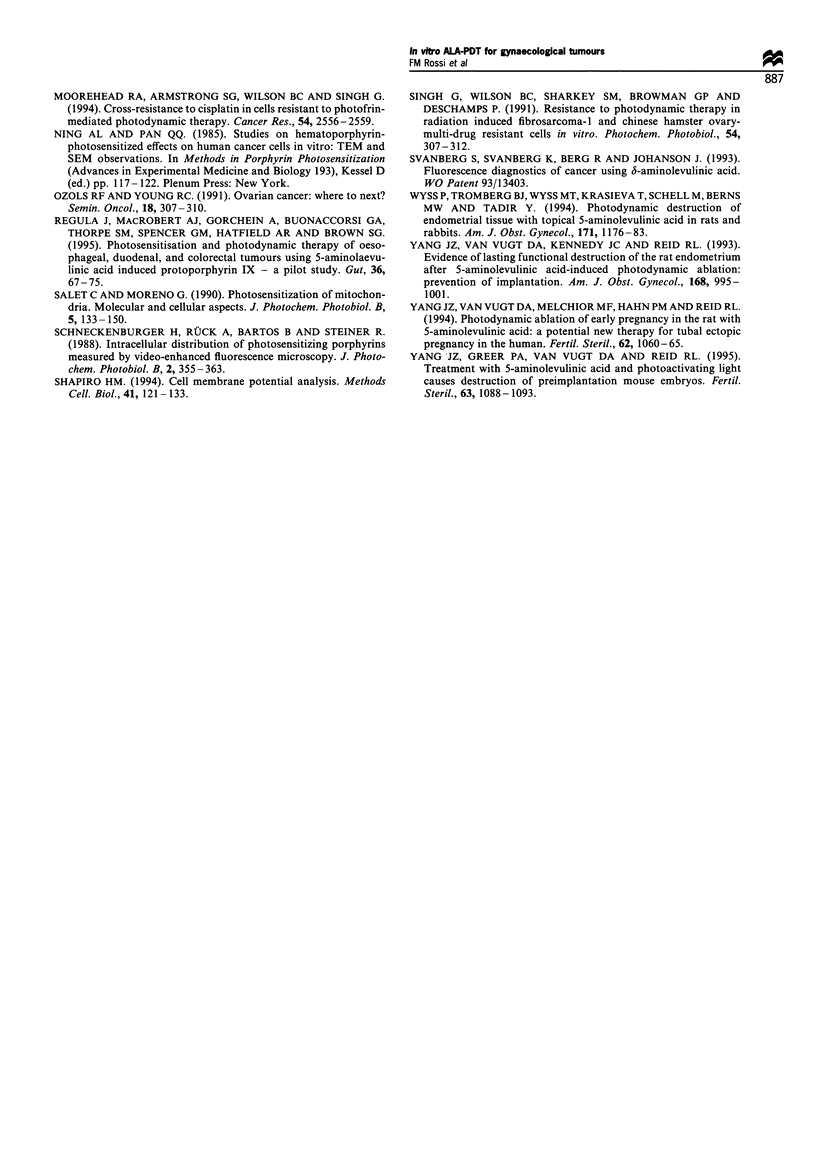# In vitro studies on the potential use of 5-aminolaevulinic acid-mediated photodynamic therapy for gynaecological tumours.

**DOI:** 10.1038/bjc.1996.452

**Published:** 1996-09

**Authors:** F. M. Rossi, D. L. Campbell, R. H. Pottier, J. C. Kennedy, E. F. Dickson

**Affiliations:** Department of Chemistry and Chemical Engineering, Royal Military College of Canada, Kingston, Ontario, Canada.

## Abstract

**Images:**


British Journal of Cancer (1996) 74, 881-887
© 1996 Stockton Press All rights reserved 0007-0920/96 $12.00

### *In vitro* studies on the potential use of 5-aminolaevulinic acid-mediated photodynamic therapy for gynaecological tumours

FM Rossi[1], DL Campbell[2], RH Pottier[1,3], JC Kennedy[1,2,4] and EF Gudgin Dickson[1]

[1]*Department of Chemistry and Chemical Engineering, The Royal Military College of Canada, Kingston, Ontario K7K 5LO, Canada; Departments of* [2]*Pathology,* [3]*Urology and* [4]*Oncology, Queen's University, Kingston, Ontario K7L 3N6, Canada.*

**Summary** Results are reported on the sensitivity of various gynaecological tumour cell lines to 5-aminolaevulinic acid-induced protoporphyrin IX-sensitised photodynamic therapy (ALA-PDT) *in vitro*. All cell lines tested accumulated ALA-induced protoporphyrin IX (PpIX) and demonstrated good sensitivity to ALA-PDT. Localisation of PpIX in the mitochondria was demonstrated by fluorescence microscopy. Subcellular damage following ALA-PDT was observed using transmission electron microscopy. This damage was localised initially to the mitochondria, with damage to membranes and the nucleus and complete loss of intracytoplasmic organisation being observed subsequently. There was no apparent difference in ALA-PDT response between a multidrug-resistant ovarian carcinoma cell line and its parent line. These results indicate that ALA-PDT has potential for application to therapy of gynaecological malignancies.

**Keywords:** protoporphyrin IX; gynaecological cancer; photodynamic therapy; fluorescence microscopy; 5-aminolaevulinic acid

Gynaecological malignancies represent a challenge to the medical community. The incidence of preinvasive squamous carcinoma has risen dramatically over the past decade and it has been predicted that mortality caused by cervical carcinoma will increase 20% by the year 2000 unless significant improvements in screening techniques are achieved (Beral and Booth, 1986). While the response to surgery, radiotherapy and chemotherapy is relatively good for early stages of the disease, there is a high mortality rate in advanced, multifocal or recurrent cancers (Corti *et al.*, 1987; Cannistra, 1993). The vast majority of patients with advanced ovarian cancer die of the disease (Ozols and Young, 1991), with overall survival rates not improving over the last 20 years. Further, the current clinical approaches to treatment are not suitable for all patients, especially for elderly women.

Photodynamic therapy (PDT) is an alternative approach to cancer treatment that has, in the last 20 years, yielded very encouraging results (Dougherty and Marcus, 1992). This technique involves the administration of a non-toxic dose of a photosensitising compound that is selectively retained by malignant tissues. Light in the visible range of the electromagnetic spectrum is then used to activate the drug *in situ*. This activated drug initiates a series of chemical reactions that leads to the selective destruction of the malignant tissues. This promising new treatment modality has already been used clinically for the experimental detection and treatment of various types of tumour, including bladder, oesophagus, skin, lung and gynaecological malignancies (Dougherty and Marcus, 1992). The combination of surgery with PDT offers a number of advantages when compared with chemotherapy. No intrinsic resistance to PDT, general toxicity or cumulative toxicity has been observed to date. The short half-life of the cytotoxic species and the mechanism of action of PDT ensure that the damage is localised primarily to the malignant tissues, sparing adjacent normal tissues. There is no reported risk of mutagenesis. PDT generally requires fewer treatments than radiation therapy, is cost-effective and is relatively non-invasive (Levy, 1994). It can be performed following debulking of large tumours during excisional surgery.

Correspondence: EF Gudgin Dickson
Received 25 October 1995; revised 2 April 1996; accepted 10 April 1996

The clinically most widely used photochemotherapeutic reagent to date has been photofrin (PF, porfimer sodium[R], photofrin II), along with several similar preparations such as dihaematoporphyrin ether (DHE), haematoporphyrin derivative (HpD) and polyhaematoporphyrin (PHP). Phase III studies in the USA with PF have focused on obstructive tumours of lung and oesophagus (Levy, 1994). The use of PDT in gynaecological lesions has provided encouraging results in preliminary studies (Corti *et al.*, 1987; DeLaney *et al.*, 1993 and references therein). For example, DeLaney reports phase I clinical trials using combined debulking surgery and DHE-PDT for disseminated intraperitoneal tumours. In Japan, PF has been used on cervical carcinoma *in situ* with a 94% complete response rate (Kato *et al.*, 1993). To date, PF has been approved for photochemotherapeutic treatment of the following malignancies: cervical (Japan), superficial bladder (Canada), oesophageal (Canada and The Netherlands), stomach (Japan) and lung (Japan and The Netherlands).

PF-PDT shows a useful degree of specificity for many malignant tissues, but the slow clearance rate of PF from normal tissues leads to prolonged skin photosensitivity (several weeks to several months). It is necessary therefore to wait at least several weeks between treatments to permit drug clearance and maintain the original level of tissue specificity. Current protocols for PF are limited to a total of three treatments. The search for other photosensitisers with more suitable properties is ongoing (Dougherty and Marcus, 1992).

An effective alternative to PF-PDT involves the use of the endogenous photosensitiser protoporphyrin IX (PpIX) (Kennedy and Pottier, 1992), which is induced to accumulate by administration of its metabolic precursor 5-aminolaevulinic acid (ALA). Administration of ALA bypasses the feedback control system in the haem biosynthetic pathway, resulting in cellular/tissue accumulation of photosensitising amounts of PpIX. Although PpIX biosynthesis is an active pathway in all nucleated cells, preferential formation/accumulation of PpIX has been demonstrated in malignant tissues (Batlle, 1993; Kennedy and Pottier, 1994). ALA-PDT has been used previously on a variety of normal and malignant cell types *in vitro* (Malik and Lugaci, 1987; Iinuma *et al.*, 1994), with a significant response to PDT by the malignant cells and lower response by the normal cells. Therapeutic application of ALA-PDT to skin and gastro-intestinal malignancies has given very encouraging preliminary results (Kennedy and Pottier, 1994; Cairnduff et al., 1994; Gossner et al., 1995; Lang et al., 1995; Lui et al., 1995; Mlkvy et al., 1995; Regula et al., 1995).

Previously there have been few reports of gynaecological applications of ALA-PDT. It has been used for endometrial ablation and to prevent embryonic implantation in laboratory animals (Yang et al., 1993, 1994, 1995; Wyss et al., 1994). ALA-PDT has been used clinically in the treatment of ovarian carcinoma metastatic in the vaginal vault with satisfactory debulking of the tumour (JC Kennedy, unpublished observations). In this study, we have determined the *in vitro* sensitivity of cell lines originating from cervical, ovarian and mammary tumours in order to provide information on the potential suitability of ALA-PDT for gynaecological tumours.

### Materials and methods

### *Cell lines*

HeLa cells (cervical epithelioid carcinoma), SiHa cells (cervical squamous carcinoma) and MDA-MB-231 cells (mammary adenocarcinoma) were obtained from the ATCC Collection Facilities (Bethesda, MD, USA). A2780-9S (ovarian carcinoma) and $2780^{AD}$ cells (a doxorubicin-resistant variant of A2780-9S) were obtained from Dr RF Ozols (NCI Bethesda, MD, USA) through Dr SPC Cole (Queen's University, Kingston, Canada). All cell lines were maintained in 25 ml tissue culture flasks (Corning, NY, USA) at 37°C and 5% carbon dioxide, in RPMI-1640 medium (Gibco, Burlington, Ontario, Canada), supplemented with 10% fetal bovine serum (FBS) (Gibco) and 1% glutamine. Cells were subcultured when confluent using 0.5% Trypsin–10% ethylenediaminetetraacetic acid (EDTA) in phosphate-buffered saline (PBS), pH 7.4, to release the cells. All chemicals and solvents were reagent grade.

### *ALA incubation*

For this and all subsequently described protocols, cells were plated close to confluency ($2 \times 10^6$ cells $ml^{-1}$) in RPMI (with 10% FBS) in 3 cm diameter wells of a six-well Falcon tissue culture plate (Becton Dickinson, Lincoln Park, NJ, USA), and left to attach overnight. Cells were then incubated in the dark in RPMI (no FBS or phenol red) with freshly prepared 5-aminolaevulinic acid hydrochloride (ALA) (Fluka Chemical Corp., Rankonkoma, NY, USA) at concentrations between 0 and 10 mM for 0 to 28 h at 37°C. For all subsequent handling, great care was taken to avoid exposing the cells to unwanted light.

### *Measurement of PpIX accumulation*

Following ALA incubation, cells were removed from the cell culture wells by vigorous pipetting, pelleted by centrifugation (2000 r.p.m. for 6 min), and resuspended in 350 μl of RPMI in 5 ml polystyrene flow cytometry tubes (Falcon). Accumulation of protoporphyrin IX (PpIX) fluorescence induced by ALA was assessed by an EPICS Elite flow cytometer (Coulter Electronics, Burlington, Ontario, Canada) as previously described (Campbell et al., 1996). This method allowed the individual examination of the fluorescence emitted by several thousands of cells ($n > 5000$). The 488 nm line of an argon ion laser was used as an excitation source (at a power of 100 mW), and a 630/20 nm bandpass (BP) (20 nm full width half maximum) filter was placed on the emission side in order to isolate the PpIX fluorescence signal. DNA-Check beads (Coulter Electronics) were used to standardise the system and minimise variability between experiments. The autofluorescence of the control sample was subtracted from the mean fluorescence intensity of each sample and these values were expressed in arbitrary fluorescence units. The data were not corrected for differences in cell volume as all of the cell lines had similar volumes as assessed by the measurement of the forward scatter on the flow cytometer (Campbell et al., 1996).

### *Spectroscopy*

Cell samples were incubated with ALA and pelleted as described above. Fluorescence emission and excitation spectra were obtained from the pellets, using a fluorimetry system described elsewhere (Dickson et al., 1995). To obtain emission spectra, excitation light from a Xe lamp filtered through a 410/10 nm BP filter was directed through one arm of a bifurcated fibre bundle onto the sample. The fluorescence was collected through the other arm of the bifurcated fibre bundle and sent to a linear diode array spectrophotofluorometer which recorded the entire emission spectrum through a 535 nm long pass (LP) filter. To obtain excitation spectra, a filter was placed on the excitation side which passed 400–650 nm; emission through a 710/10 nm BP filter was quantified while scanning the excitation wavelength, using a stepping monochromator, every 5 nm between 350 and 670 nm. The excitation spectra were corrected for excitation intensity.

### *Cell photosensitisation*

Cells, incubated with ALA as previously described, were light-treated with a tungsten lamp equipped with a heat filter and a 600–700 nm BP filter. The photosensitising light was focused perpendicularly on the cell culture well, which was placed on a rotating holder to ensure uniformity of irradiation (irradiance $70 \pm 10$ mW $cm^{-2}$), determined using an apertured calorimetric detector (Sciencetech 380101, Boulder, CO, USA). After a predetermined time of light exposure, cells were collected and labelled with fluorescent dyes in order to assess cell viability by flow cytometry as described elsewhere (Campbell et al., 1996). In brief, cells were labelled using the Live–Dead Eukolite Viability/Cytotoxicity Kit (L-3224, Molecular Probes Inc., Eugene OR, USA). This kit uses two fluorescent dyes to assess vital functions. One dye, calcein AM, is converted by intracellular esterases to calcein, which fluoresces at 535 nm and is retained by viable cells. The other dye, ethidium homodimer-1, penetrates damaged membranes of dead or dying cells, binds to nucleic acids and fluoresces at 640 nm. RPMI (100 μl) containing ethidium homodimer-1 (4 μM) and calcein AM (2 μM) were added to each cell sample. Cells were then incubated for 30 min in the dark at 37°C before the flow cytometric measurements. Specific fluorescence was detected by the use of a 525/30 nm BP filter (for calcein) and a 595 nm LP filter (for ethidium). This technique permits one to gate electronically on whole single cells (excluding debris and clumps), using forward scatter *vs* side scatter information.

### *Fluorescence microscopy*

Fluorescence microscopic studies were performed using a system that combines a cooled charge-coupled device (CCD) camera imaging system with either a Leitz Aristoplan epifluorescence microscope or a Meridian confocal microscope. Such a system permits switching from a cytological to a fluorescent image of the same cells. The epifluorescence microscope was equipped with a 100 W mercury lamp filtered with an I3 filter cassette (excitation 450–490 nm, dichroic 510 nm, barrier filter 520 nm LP). The light source for the confocal microscope was the 488 nm line of an argon ion laser, and the microscope was equipped with a filter wheel containing eight selections for filtering the emission light. All images were captured using a cooled CCD camera (Meridian Instruments, Okemos, MI, USA), and were digitised using a MCID-M4 microcomputer system (Imaging Research, St. Catherine's, Ontario, Canada). Subconfluent cells were incubated on glass coverslips in the presence of ALA or media under the conditions described above. Coverslips were then mounted on a slide and observed with a $100 \times$ oil immersion objective lens through a 620/40 nm BP filter to observe PpIX fluorescence. For the mitochondrial colocalisation studies, the cells on the coverslips were further incubated for 10 min in 10 $\mu$g ml$^{-1}$ rhodamine 123 in RPMI (without phenol red), rinsed in RPMI without phenol red, then observed as previously described through a 525/30 nm BP filter. The no ALA and no rhodamine controls exhibited no cross-fluorescence under the experimental conditions used.

### *Electron microscopy*

Cells were incubated with ALA, irradiated (when required) and collected as previously described. Cells were centrifuged (1 min, 12 000 r.p.m.), fixed in 2% paraformaldehyde–0.5% glutaraldehyde in 0.2 M cacodylate buffer, post-fixed in 1% osmium in 0.1 M cacodylate buffer for 1 h, dehydrated in ethanol and embedded in epon 812 (epoxy resin). Thin sections (70 nm) were stained with uranyl acetate and lead citrate and then observed in a Hitachi H500 transmission electron microscope (TEM).

### Results

### *ALA-induced accumulation of PpIX*

Figure 1 shows that the emission and excitation spectra observed from the A2780-9S cell pellets correspond to those of PpIX. No fluorescence emission spectrum characteristic of other endogenous porphyrins was observed. Similar results were obtained for all of the cell lines studied.

After dark incubation with ALA, all the cell lines studied showed some degree of porphyrin accumulation, with a broad histogram of fluorescence intensities. Figure 2 shows that the accumulation of PpIX, as judged by the PpIX fluorescence intensity, increased with the time of dark incubation, and tended towards a plateau after 5 h for most cell lines. However, in no case was an actual plateau reached for incubation times up to 28 h. Only the A2780-9S cells appear to accumulate PpIX at linearly increasing concentrations for up to 28 h.

Figure 3 shows that at concentrations of ALA lower than 2.5 mM, PpIX accumulation appeared to be dose dependent, but all cell lines showed an accumulation plateau for concentration of ALA greater than 5 mM. At higher ALA doses (10 mM) there was, for the A2780-9S and SiHa cells, a small decrease in accumulation, possibly owing to the acidity of the ALA solution exceeding the buffer capability of the medium. Based on the above results, a concentration of ALA corresponding to the plateau region of the PpIX accumulation *vs* ALA concentration curves (5 mM) was used for all further experiments.

### *ALA-induced photosensitisation*

After incubation with ALA, the cells became very sensitive to light-induced damage. Figure 4 shows that the observed multilogarithmic killing was very rapid and directly dependent on the amount of energy delivered. Under these experimental conditions, there were no significant differences in the amount of cell killing among different cell lines. Light treatment alone did not kill the cells. No decrease in ALA-PDT killing efficacy was observed for the doxorubicin-resistant cells (2780$^{AD}$) relative to the non-resistant equivalent. Cell kill was dependent upon the duration of dark incubation with ALA before irradiation, that is, on the amount of PpIX that had accumulated. Incubation of cells in ALA for 20 h resulted in more photosensitisation than incubation for 5 h.

### *PpIX localisation*

Subcellular localisation of PpIX was studied by fluorescence microscopy. Figure 5a shows the very distinctive intracellular PpIX fluorescence distribution with a punctate pattern in the perinuclear region. The extranuclear distribution of the PpIX fluorescence was confirmed by comparing the images of the same cells taken in bright field and epifluorescence modes. The punctate pattern of the fluorescence indicated a subcellular organelle distribution. Figure 5b shows that, after co-incubating the cells with ALA and rhodamine 123, the same intracellular fluorescence pattern could be observed for both stains, indicating that PpIX is accumulating in the

**Figure 1** Characteristic protoporphyrin IX excitation (**a**) and emission (**b**) spectra obtained from pelleted A2780-9S cells incubated with 5 mM ALA in the dark for 5 h.

**Figure 2** Protoporphyrin IX accumulation as a function of time of incubation of cells in the dark with 5 mM ALA. (○) HeLa cells, (●) A2780-9S cells, (△) 2780$^{AD}$ cells, (■) SiHa cells, (▼) MDA-MB-231 cells. Each data point represents the average of at least three determinations. The standard deviation was less than 15%.

**Figure 3** Protoporphyrin IX accumulation obtained after 12 h of dark incubation of cells, as a function of ALA concentration. (○) HeLa cells, (●) A2780-9S cells, (△) 2780$^{AD}$ cells, (■) SiHa cells, (▼) MDA-MB-231 cells. Each data point represents the average of at least three determinations, with a standard deviation less than 15%.

mitochondria [Rhodamine is a known stain for mitochondria (Shapiro, 1994)].

The location of subcellular phototoxic damage was studied by transmission electron microscopy. TEM pictures taken after cell incubation with ALA and different doses of light showed diffuse mitochondrial alterations as an early phenomenon (Figure 6), with mitochondria swollen and showing enlarged intercristae spaces. At higher light doses (Figure 7), vacuolisation and vesiculation of the cytoplasm, condensation of the nuclear chromatin, swelling of the nuclear membrane and rounding of the cellular shape were observed. At even later stages the integrity of the cytoplasmic membrane and the cytoplasmic structure was lost and the nuclei appeared pycnotic.

### Discussion

Results obtained in this *in vitro* study clearly show that cell lines derived from gynaecological tumours can accumulate ALA-induced PpIX, exhibiting a significant degree of PpIX fluorescence. There is a significant correlation between ALA incubation time, that is, the amount of PpIX accumulation, and efficacy of ALA-PDT, as judged by the survival curves. A minimum of three logs of cell kill can be obtained by ALA-PDT at doses $>15$ J cm$^{-2}$ of red light for larger amounts of accumulated PpIX. Although *in vitro* work on cell lines cannot be extrapolated directly to *in vivo* systems, these results, along with other preliminary results obtained in our laboratory on surgical specimens, indicate that ALA-induced PpIX may be useful in the detection and treatment of gynaecological tumours. ALA-PDT has been used to treat an ovarian carcinoma metastatic in the vaginal vault, which showed a good clinical response with adequate tissue specificity (JC Kennedy, unpublished results).

For detection purposes, HpD fluorescence has been a useful tool, showing good correlation with histological findings in delineating the limits of cervical lesions (Gray *et al.*, 1967; Kyriazis *et al.*, 1973). PpIX fluorescence has been used in the same manner, for example in the bladder (Svanberg *et al.*, 1993; Kriegmair *et al.*, 1994), and should find clinical usefulness in detection of metastatic gynaecological tumours also.

Drug resistance is a serious problem for conventional chemotherapy and has been reported also for PDT using photofrin in tissue culture. Cell lines resistant to PF-PDT have also shown cross-resistance to other therapeutic methods. Chinese hamster ovary fibroblast multidrug-resistant (CHO-MDR) cells have shown cross-resistance to PF-PDT (Singh *et al.*, 1991; Mitchell *et al.*, 1988). In this case

**Figure 4** Survival of cells incubated with 5 mM ALA for 5 h (▲) and 20 h (◆) as a function of light dose. Each data point represents the average of at least three determinations, with a standard deviation less than 15%. There was no significant cell death following light treatment alone.

**Figure 5** Characteristic (**a**) PpIX and (**b**) rhodamine fluorescence distributions in SiHa cells incubated 20 h with 5 mM ALA, and then for 10 min with rhodamine 123. Photographs were taken through a 620 nm BP filter (for PpIX) and 530 nm BP filter (for rhodamine).

**Figure 6** MDA-MB-231 cells incubated for 20 h with 5 mM ALA. (**a**) Control cell (no light); (**b**) cell exposed to 4.2 J $cm^{-2}$ light dose; (**c**) mitochondria of control cell; (**d**) mitochondria of exposed cell. Note swelling of mitochondria, loss of microvilli and condensation of nuclear chromatin after light exposure.

**Figure 7** $2780^{AD}$ cells incubated for 20 h with 5 mM ALA. (**a**) Control cell (no light); (**b**) cell exposed to 6.3 J $cm^{-2}$ light dose; (**c**) mitochondria of control cell; (**d**) mitochondria of exposed cell. Note in the light-exposed cells the marked swelling of the mitochondria with enlargement of intercristae space, vacuolisation of cytoplasm, rupture of cytoplasmic membrane and loss of cytoplasmic structure, swelling of the nuclear membrane and nuclear pycnosis. These phenomena were seen in all cell types examined.

multidrug resistance conferred some degree of PF-PDT resistance but the converse was not true. In contrast, a MDR human breast cancer line was not PF-PDT resistant (Mitchell *et al*., 1988). PF-PDT-resistant radiation-induced fibrosarcoma (RIF) cells have shown cross-resistance to cisplatin, but do not exhibit multidrug resistance (Moorehead *et al*., 1994). Mouse tumour cells resistant to PF-PDT also did not exhibit a multidrug resistance phenotype nor did they have altered porphyrin uptake properties (Luna and Gomer, 1991). Explanations for these various observations include altered subcellular localisation of photosensitiser, photodamage repair mechanisms or plasma and mitochondrial membrane potentials, with different mechanisms existing in general for PF-PDT- and multidrug-induced resistance. Possible mechanisms for drug resistance in general include, for example, altered drug transport (implicated in multidrug resistance), increased metallothionein or glutathione levels, altered DNA adduct formation and/or repair and altered membrane potentials. For ALA-PDT, our data indicate that there is no significant difference in response between the doxorubicin- resistant (MDR) ovarian cell line $2780^{AD}$ and its doxorubicin-sensitive parental cell line. Thus, at least in these cells, multidrug resistance does not appear to confer any resistance to ALA-PDT. We have also demonstrated that cisplatin-resistant small-cell lung cancer cells do not show resistance to ALA-PDT (Campbell *et al*., 1995).

The mechanisms for accumulation and the sites of phototoxic damage are different for ALA-induced PpIX (this study and Iinuma *et al*., 1994) and PF (Schneckenburger *et al*., 1988; Berns *et al*., 1982; Moan *et al*., 1989; Salet and Moreno, 1990; Kessel, 1986). PF passes from the extracellular environment into the plasma membrane, and from there is believed to migrate to various intracellular sites including the nuclear and mitochondrial membranes. Various transport processes must be involved and sites of action can be different depending on the delivery method. On the other hand, since the PpIX is synthesised largely in the mitochondria (Batlle, 1993), no PpIX transport is required before accumulation in the location sensitive to damage. Mitochondrial alterations suggest that this organelle is in fact a primary site of ALA-PDT damage. TEM results obtained in this study are similar to those previously reported on cells incubated with haematoporphyrin (Milanesi *et al*., 1989; Malik *et al*., 1992), ALA (Malik and Lugaci, 1987) and PF (Evensen *et al*., 1988; Leunig *et al*., 1994; Ning and Pan, 1985). Observations common to all cell lines studied included swollen mitochondria, loss of microvilli and vacuolisation and vesiculation of the cytoplasm. Also observed are the enlargement of the nuclear membrane, probably owing to influx of water in the inner space as the two membranes are still connected at the level of the nuclear pores, and rupture of the plasma membrane leading to complete loss of intracytoplasmic organisation. Nuclear alterations included condensation of the chromatin and a pycnotic appearance of the nuclei. From the flow cytometric data obtained at various light doses (not shown), it can be seen that at low doses of light, early damage is manifested as increased permeability of the plasma membrane (decrease in the calcein fluorescence) without a concomitant increase in ethidium fluorescence; at higher light doses nuclear membrane permeability increases (increase in ethidium fluorescence). This suggests that nuclear alterations are the consequence of an indirect process, which may imply a $Ca^{2+}$ imbalance resulting in activation of endonucleases.

While several questions on the detailed mechanism of ALA-PDT mode of action still remain to be solved, our results indicate that ALA induces the accumulation of fluorescing and photosensitising concentrations of PpIX in a panel of malignant gynaecological cell lines. Studies are planned to evaluate the clinical potential of this phenomenon. To date, good clinical responses have been seen in preliminary patient studies of ALA-PDT in breast secondaries in the chest wall (Kennedy and Pottier, 1992) and in cervical and ovarian carcinoma involving the vaginal vault (JC Kennedy, unpublished observations).

**Abbreviations**

ALA, 5-aminolaevulinic acid; PpIX, protoporphyrin IX; PDT, photodynamic therapy; PF, Photofrin II; DHE, dihaematoporphyrin ether; HpD, haematoporphyrin derivative; PHP, polyhaematoporphyrin; PBS, phosphate-buffered saline; BP, bandpass; BW, bandwidth; LP, long pass; TEM, transmission electron microscope; MDR, multidrug resistant; RIF, radiation-induced fibrosarcoma; CHO, chinese hamster ovary; CCD, charge-coupled device; EDTA, ethylenediaminetetraacetic acid; FCS, fetal calf serum.

**Acknowledgements**

FR acknowledges support from a Government of Canada Visiting Scientist Fellowship, DC from Ontario Graduate Scholarships, RP from the Department of National Defence Canada, JCK from Draxis Pharmaceuticals Inc., Medical Research Council of Canada and Ontario Cancer Treatment and Research Foundation.

### References

BATLLE AMC. (1993). Porphyrins, porphyrias, cancer and photodynamic therapy – a model for carcinogenesis. *J. Photochem. Photobiol. B*, **20**, 5–22.

BERAL V AND BOOTH M. (1986). Prediction of cervical cancer incidence and mortality in England and Wales. *Lancet*, **1**, 495.

BERNS MW, DAHLMAN A, JOHNSON FM, BURNS R, SPERLING D, GUILTINAN M, SIEMENS A, WALTER R, WRIGHT W, HAMMER-WILSON M AND WILE A. (1982). *In vitro* cellular effects of hematoporphyrin derivative. *Cancer Res.*, **42**, 2325–2329.

CAIRNDUFF F, STRINGER MR, HUDSON EJ, ASH DV AND BROWN SB. (1994). Superficial photodynamic therapy with topical 5-aminolaevulinic acid for superficial primary and secondary skin cancer. *Br. J. Cancer*, **69**, 605–608.

CAMPBELL DL, ROSSI FM, POTTIER RH AND KENNEDY JC. (1995). Physiological factors affecting ALA-induced protoporphyrin IX photosensitization *in vitro*. *Photochem. Photobiol.*, **61**, 94S (abstract WPM-D3, *23rd Annual Meeting of Am. Soc. Photobiol.*, Washington, DC).

CAMPBELL DL, FISHER ME, JOHNSON JG, ROSSI FM, CAMPLING BG, POTTIER RH AND KENNEDY JC. (1996). Flow cytometric technique for quantitating cytotoxic response to photodynamic therapy. *Photochem. Photobiol.*, **63**, 111–116.

CANNISTRA SA. (1993). Cancer of the ovary. *N. Engl. J. Med.*, **329**, 1550–1559.

CORTI L, TOMIO L, MALUTA S, MINUCCI D, FONTANA M, CITTAR H AND CALZAVARA A. (1987). Recurring gynaecologic cancer treatment with photodynamic therapy. *Photochem. Photobiol.*, **46**, 949–952.

DELANEY TF, SINDELAR WF, TOCHNER Z, SMITH PD, FRIAUF WS, THOMAS G, DACHOWSKI L, COLE JW, STEINBERG SM AND GLATSTEIN E. (1993). Phase I study of debulking surgery and photodynamic therapy for disseminated intraperitoneal tumors. *Int. J. Radiat. Oncol. Biol. Phys.*, **25**, 445–457.

DICKSON EFG, HOLMES H, JORI G, KENNEDY JC, NADEAU P, POTTIER RH, ROSSI F, RUSSELL DA AND WEAGLE GE. (1995). On the source of the oscillations observed during *in vivo* zinc phthalocyanine fluorescence pharmacokinetic measurements in mice. *Photochem. Photobiol.*, **61**, 506–509.

DOUGHERTY TJ AND MARCUS SL. (1992). Photodynamic therapy. *Eur. J. Cancer*, **28A**, 1734–1742.

EVENSEN JF, MALIK Z AND MOAN J. (1988). Ultrastructural changes in the nuclei of human carcinoma cells after photodynamic treatment with haematoporphyrin derivative and tetrasodium-meso-tetra- (4-sulphonatophenyl)porphine. *Lasers Med. Sci.*, **3**, 195–206.

GOSSNER L, SROKA R, HAHN EG AND ELL C. (1995). Photodynamic therapy: successful destruction of gastrointestinal cancer after oral administration of aminolevulinic acid. *Gastrointestinal Endoscopy*, **41**, 55–58.

GRAY MJ, LIPSON R, MAECK JVS, PARKER L AND ROMEYN D. (1967). Use of hematoporphyrin derivative in detection and management of cervical cancer. *Am. J. Obst. Gyn.*, **99**, 766–771.

IINUMA S, FARSHI SS, ORTEL B AND HASAN T. (1994). A mechanistic study of cellular photodestruction with δ-aminolevulinic acid-induced porphyrin. *Br. J. Cancer*, **70**, 21–28.

KATO H, HORAI T, FURUSE K, FUKUOKA M, SUZUKI S, HIKI Y, ITO Y, MIMURA S, TENJIN Y AND HISAZUMI H. (1993). Photodynamic therapy for cancers: a clinical trial of porfimer sodium in Japan. *Jpn. J. Cancer Res.*, **84**, 1209–1214.

KENNEDY JC AND POTTIER RH. (1992). Endogenous protoporphyrin IX, a clinically useful photosensitizer for photodynamic therapy. *J. Photochem. Photobiol. B*, **14**, 275–292.

KENNEDY JC AND POTTIER RH. (1994). Using δ-aminolevulinic acid in cancer therapy. In *Porphyric Pesticides: Chemistry, Toxicology, and Pharmaceutical Applications*, Duke SO, Rebeiz CA, (eds), ACS Symposium Series No. 559, pp. 291–301. American Chemical Society: Washington.

KESSEL D. (1986). Sites of photosensitization by derivatives of hematoporphyrin. *Photochem. Photobiol.*, **44**, 489–493.

KRIEGMAIR M, BAUMGARTNER R, KNUECHEL R, STEINBACK P, EHSAN A, LUMPER W, HOFSTADTER F AND HOFSTETTER A. (1994). Fluorescence photodetection of neoplastic urothelial lesions following intravesical instillation of 5-aminolevulinic acid. *Urology*, **44**, 836–841.

KYRIAZIS GA, BALIN H AND LIPSON RL. (1973). Hematoporphyrin-derivative-fluorescence test colposcopy and colpophotography in the diagnosis of atypical metaplasia, dysplasia, and carcinoma *in situ* of the cervix uteri. *Am. J. Obst. Gyn.*, **117**, 375–380.

LANG S, BAUMGARTNER R, STRUCK R, LEUNIG A, GUTMANN R AND FEYH J. (1995). Photodynamic diagnosis and therapy of neoplasms of the facial skin after topical administration of 5-aminolevulinic acid (in German). *Laryngo- Rhino- Otol.*, **74**, 85–89.

LEUNIG A, STAUB F, PETERS J, LEIDERER R, FEYH J AND GOETZ A. (1994). Damage to tumour cells after photodynamic therapy (in German). *Laryngo- Rhino- Otol.*, **73**, 102–107.

LEVY JG. (1994). Photosensitizers in photodynamic therapy. *Semin. Oncol.*, **21**, (suppl. 15), 4–10.

LUI H, SALASCHE S, KOLLIAS N, FLOTTE T, MCLEAN D AND ANDERSON RR. (1995). Photodynamic therapy of nonmelanoma skin cancer with topical aminolevulinic acid: a clinical and histological study. *Arch. Dermatol.*, **131**, 737–738.

LUNA MC AND GOMER CJ. (1991). Isolation and initial characterization of mouse tumor cells resistant to porphyrin-mediated photodynamic therapy. *Cancer Res.*, **51**, 4243–4249.

MALIK Z AND DJALDETTI M. (1979). 5-aminolevulinic acid stimulation of porphyrins and hemoglobin synthesis by uninduced Friend erythrocyte leukemia cells. *Cell Different.*, **8**, 223–225.

MALIK Z AND LUGACI H. (1987). Destruction of erythroleukaemic cells by photoactivation of endogenous porphyrins. *Br. J. Cancer*, **56**, 589–595.

MALIK Z, FARAGGI A AND SAVION N. (1992). Ultrastructural damage in photosensitized endothelial cells: dependence on hematoporphyrin delivery pathways. *J. Photochem. Photobiol. B*, **14**, 359–368.

MILANESI C, SORGATO F AND JORI G. (1989). Photokinetic and ultrastructural studies on porphyrin photosensitization of HeLa cells. *Int. J. Radiat. Biol.*, **55**, 59–69.

MITCHELL JB, GLATSTEIN E, COWAN KH AND RUSSO A. (1988). Photodynamic therapy of multi-drug resistant cell lines (abstract). *Proc. Am. Assoc. Cancer Res.*, **29**, 315.

MLKVY P, MESSMAN H, REGULA J, CONIO M, PAUER M AND MILLSON CE, MACROBERT AJ, BOWN SJ. (1995). Sensitization and photodynamic therapy (PDT) of gastrointestinal tumors with 5-aminolaevulinic acid (ALA) induced protoporphyrin IX (PPIX). A pilot study. *Neoplasma*, **42**, 109–113.

MOAN J, BERG K, KVAN E, WESTERN A, MALIK Z, RÜCK A AND SCHNECKENBURGER H. (1989). Intracellular localization of photosensitizers. In *Photosensitizing Compounds: their Chemistry, Biology and Clinical Use*, Bock G, Harnett S. (eds), (Ciba Foundation Symposium 146), pp. 95–111. J Wiley & Sons: Chichester.

MOOREHEAD RA, ARMSTRONG SG, WILSON BC AND SINGH G. (1994). Cross-resistance to cisplatin in cells resistant to photofrin-mediated photodynamic therapy. *Cancer Res.*, **54**, 2556–2559.

NING AL AND PAN QQ. (1985). Studies on hematoporphyrin-photosensitized effects on human cancer cells in vitro: TEM and SEM observations. In *Methods in Porphyrin Photosensitization* (Advances in Experimental Medicine and Biology 193), Kessel D (ed.) pp. 117–122. Plenum Press: New York.

OZOLS RF AND YOUNG RC. (1991). Ovarian cancer: where to next? *Semin. Oncol.*, **18**, 307–310.

REGULA J, MACROBERT AJ, GORCHEIN A, BUONACCORSI GA, THORPE SM, SPENCER GM, HATFIELD AR AND BROWN SG. (1995). Photosensitisation and photodynamic therapy of oesophageal, duodenal, and colorectal tumours using 5-aminolaevulinic acid induced protoporphyrin IX – a pilot study. *Gut*, **36**, 67–75.

SALET C AND MORENO G. (1990). Photosensitization of mitochondria. Molecular and cellular aspects. *J. Photochem. Photobiol. B*, **5**, 133–150.

SCHNECKENBURGER H, RÜCK A, BARTOS B AND STEINER R. (1988). Intracellular distribution of photosensitizing porphyrins measured by video-enhanced fluorescence microscopy. *J. Photochem. Photobiol. B*, **2**, 355–363.

SHAPIRO HM. (1994). Cell membrane potential analysis. *Methods Cell. Biol.*, **41**, 121–133.

SINGH G, WILSON BC, SHARKEY SM, BROWMAN GP AND DESCHAMPS P. (1991). Resistance to photodynamic therapy in radiation induced fibrosarcoma-1 and chinese hamster ovary-multi-drug resistant cells *in vitro*. *Photochem. Photobiol.*, **54**, 307–312.

SVANBERG S, SVANBERG K, BERG R AND JOHANSON J. (1993). Fluorescence diagnostics of cancer using δ-aminolevulinic acid. *WO Patent* 93/13403.

WYSS P, TROMBERG BJ, WYSS MT, KRASIEVA T, SCHELL M, BERNS MW AND TADIR Y. (1994). Photodynamic destruction of endometrial tissue with topical 5-aminolevulinic acid in rats and rabbits. *Am. J. Obst. Gynecol.*, **171**, 1176–83.

YANG JZ, VAN VUGT DA, KENNEDY JC AND REID RL. (1993). Evidence of lasting functional destruction of the rat endometrium after 5-aminolevulinic acid-induced photodynamic ablation: prevention of implantation. *Am. J. Obst. Gynecol.*, **168**, 995–1001.

YANG JZ, VAN VUGT DA, MELCHIOR MF, HAHN PM AND REID RL. (1994). Photodynamic ablation of early pregnancy in the rat with 5-aminolevulinic acid: a potential new therapy for tubal ectopic pregnancy in the human. *Fertil. Steril.*, **62**, 1060–65.

YANG JZ, GREER PA, VAN VUGT DA AND REID RL. (1995). Treatment with 5-aminolevulinic acid and photoactivating light causes destruction of preimplantation mouse embryos. *Fertil. Steril.*, **63**, 1088–1093.